# Dynamic Near-Infrared Optical Imaging of 2-Deoxyglucose Uptake by Intracranial Glioma of Athymic Mice

**DOI:** 10.1371/journal.pone.0008051

**Published:** 2009-11-30

**Authors:** Heling Zhou, Kate Luby-Phelps, Bruce E. Mickey, Amyn A. Habib, Ralph P. Mason, Dawen Zhao

**Affiliations:** 1 Department of Radiology, The University of Texas Southwestern Medical Center, Dallas, Texas, United States of America; 2 Department of Cell Biology, The University of Texas Southwestern Medical Center, Dallas, Texas, United States of America; 3 Department of Neurological Surgery, The University of Texas Southwestern Medical Center, Dallas, Texas, United States of America; 4 Department of Neurology, The University of Texas Southwestern Medical Center, Dallas, Texas, United States of America; Cuban Neuroscience Center, Cuba

## Abstract

**Background:**

It is recognized that cancer cells exhibit highly elevated glucose metabolism compared to non-tumor cells. We have applied *in vivo* optical imaging to study dynamic uptake of a near-infrared dye-labeled glucose analogue, 2-deoxyglucose (2-DG) by orthotopic glioma in a mouse model.

**Methodology and Principal Findings:**

The orthotopic glioma model was established by surgically implanting U87-luc glioma cells into the right caudal nuclear area of nude mice. Intracranial tumor growth was monitored longitudinally by bioluminescence imaging and MRI. When tumor size reached >4 mm diameter, dynamic fluorescence imaging was performed after an injection of the NIR labeled 2-DG, IRDye800CW 2-DG. Real-time whole body images acquired immediately after i.v. infusion clearly visualized the near-infrared dye circulating into various internal organs sequentially. Dynamic fluorescence imaging revealed significantly higher signal intensity in the tumor side of the brain than the contralateral normal brain 24 h after injection (tumor/normal ratio, TNR  = 2.8±0.7). Even stronger contrast was achieved by removing the scalp (TNR  = 3.7±1.1) and skull (TNR  = 4.2±1.1) of the mice. In contrast, a control dye, IRDye800CW carboxylate, showed little difference (1.1±0.2). *Ex vivo* fluorescence imaging performed on ultrathin cryosections (20 µm) of tumor bearing whole brain revealed distinct tumor margins. Microscopic imaging identified cytoplasmic locations of the 2-DG dye in tumor cells.

**Conclusion and Significance:**

Our results suggest that the near-infrared dye labeled 2-DG may serve as a useful fluorescence imaging probe to noninvasively assess intracranial tumor burden in preclinical animal models.

## Introduction

Glioblastoma multiform (GBM) is a lethal intracranial cancer, which exhibits a relentless malignant progression and is highly resistant to conventional multimodal therapies. GBM is characterized by the nature of extensive infiltration into surrounding normal brain tissue, which results in incomplete tumor resection and consequent recurrence [1], [2]. It is imperative to improve diagnostic imaging to evaluate intracranial tumor growth and therapeutic responses. Optical imaging has been rapidly adapted to cancer research. Recently, optical imaging using fluorescent dye labeled tumor-specific molecules has been successfully applied to imaging glioma in preclinical animal models based on overexpression of such markers in glioma [3]–[5]. In the clinic, several recent studies have demonstrated the ability of fluorescence imaging to facilitate radical resection of GBM during surgery [6]–[8]. Promising results reported by Stummer, *et al.* have shown that gross total resection of glioma guided by intraoperative fluorescence imaging is associated with improved prognosis of the patients with a median survival of 15–18 months, compared to 10–12 months after a subtotal resection or about 6 months after biopsy alone [8].

It is well recognized that cancer cells exhibit highly elevated glucose metabolism and up-regulated glucose transporters (GLUTs) compared to non-tumor cells. On this basis, ^18^FDG, the glucose analogue, has been used as the most common PET radiotracer to visualize clinical tumors and their metastases. However, a drawback of ^18^FDG PET for brain tumors is strong background signals of normal brain tissues, which often compromise the ability to diagnose the brain tumors. Moreover, PET has a low spatial resolution in spite of high sensitivity. Optical imaging by visualizing fluorescently labeled tumor cells has recently emerged as an attractive approach to facilitate identification of infiltrative tumors and sentinel lymph node metastases [9]–[14]. Alternative to radioactive deoxyglucose, fluorescent derivatives of 2-DG, *e.g.*, 2-[*N*-(7-nitrobenz-2-oxa-1,3-diazol-4-yl)amino]-2-deoxy-D-glucose (2-NBDG) have shown a greater tendency to be delivered and trapped in tumor cells [15], [16]. However, the short wavelength of 2-NBDG (excitation, 475 nm; emission, 550 nm) limits its applications for *in vivo* imaging. Near infrared fluorescence has several advantages over the use of visible fluorophores including deeper penetration due to less tissue absorption and scattering of light, and minimal autofluorescence. IRDye800CW 2-DG (Li-Cor Bioscience), a NIR dye conjugated with 2-deoxyglucose (peak excitation 785 nm, emission 810 nm), has recently been developed and demonstrated as a tumor-targeting optical contrast agent in various tumors implanted subcutaneously in mice, which can be visualized *in vivo* by fluorescence imaging [17], [18]. Moreover, a recent study of pharmacokinetics has shown that there is essentially no retention of the dye in normal mouse brain 24 h after injection [19]. Thus, the 2-DG NIR dye may serve as an ideal contrast agent for optically imaging brain tumors.

In this study, we first applied bioluminescence imaging (BLI) and MRI to assess non-invasively intracranial tumor growth in an orthotopic glioma model in nude mice. We then exploited IRDye800CW 2-DG for dynamic *in vivo* imaging of these brain tumors. *In vivo* observations were validated by both *ex vivo* fluorescence imaging of cryosections of tumor bearing brain tissues and histological staining. Finally, fluorescence microscopic studies were performed to identify locations of the 2-DG dye in tumors and normal brain.

## Results

**Figure 1:**
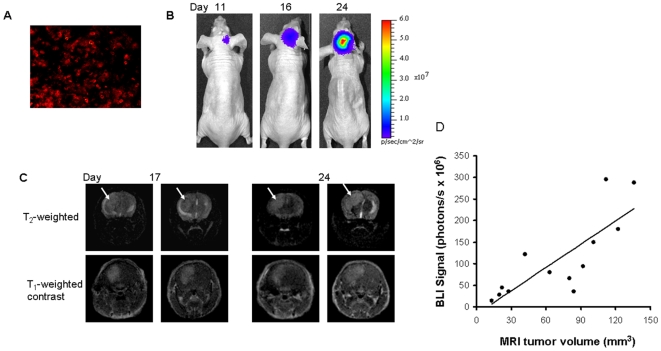
BLI and MRI monitoring of intracranial growth of U87 glioma in a mouse. **A**. Immunostaining with anti-luciferase antibody showed extensive expression of luciferase in tumor tissues. **B**. BLI showed increasing light intensity in the mouse brain over time. **C**. Transaxial MRI detected an intracranial tumor on consecutive slices (arrows) based on both T_2_- weighted and T_1_-weighted contrast enhanced MRI, which was found to grow in size on the follow-up study (84 mm^3^ on day 17, 136 mm^3^ on day 24). **D**. A good correlation between the BLI signal and actual intracranial tumor volume was observed (r = 0.8, p<0.05).

Immunohistochemical study showed extensive expression of luciferase in the U87-luc cells of intracranial tumor tissues (Fig. 1A). Longitudinal BLI studies revealed a weak signal initially 11 days after tumor implantation, which became stronger on follow-up to day 24 (Fig. 1B). The mean light intensity detected on day 24 for the group of tumor bearing mice was significantly greater than on day 11 (16×10^7^ versus 11×10^6^ photons/s; p<0.05). MRI studies based on T_2_-weighted and T_1_-weighted contrast enhanced images confirmed an intracranial tumor located in the right side of the brain of each animal (Fig. 1C). A significant correlation was found between BLI signal and actual tumor volume measured by MRI (r = 0.8, p<0.05; Fig. 1D). Tumor volume determined on the last MRI scan (one day before the *in vivo* fluorescence imaging) varied from 42 to 136 mm^3^ in the seven animals. There was no significant difference in tumor volume between the group receiving the 2-DG dye or the control dye when the *in vivo* fluorescence imaging was performed (p = 0.5).

**Figure 2:**
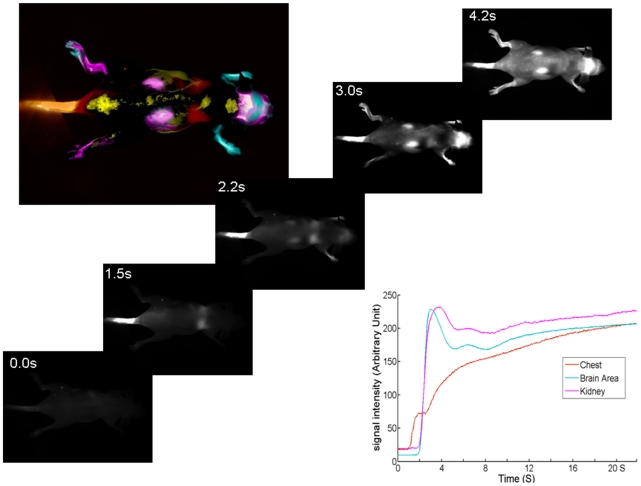
*In vivo* real-time near infrared imaging of a mouse. A series of whole body images was acquired before and after injection of near infrared dye labeled 2-DG, IRDye800CW 2-DG, via a mouse tail vein. Selected images captured the first pass perfusion of the dye into various internal organs: heart and lung area at 1.2 s, brain and upper limbs at 2.5 s, kidney at 4.5 s, etc. Principal Component Analysis of the kinetics was used to identify tissue regions based on DyCE software as shown in the color presentation (top left) and representative time courses (bottom right).

**Figure 3:**
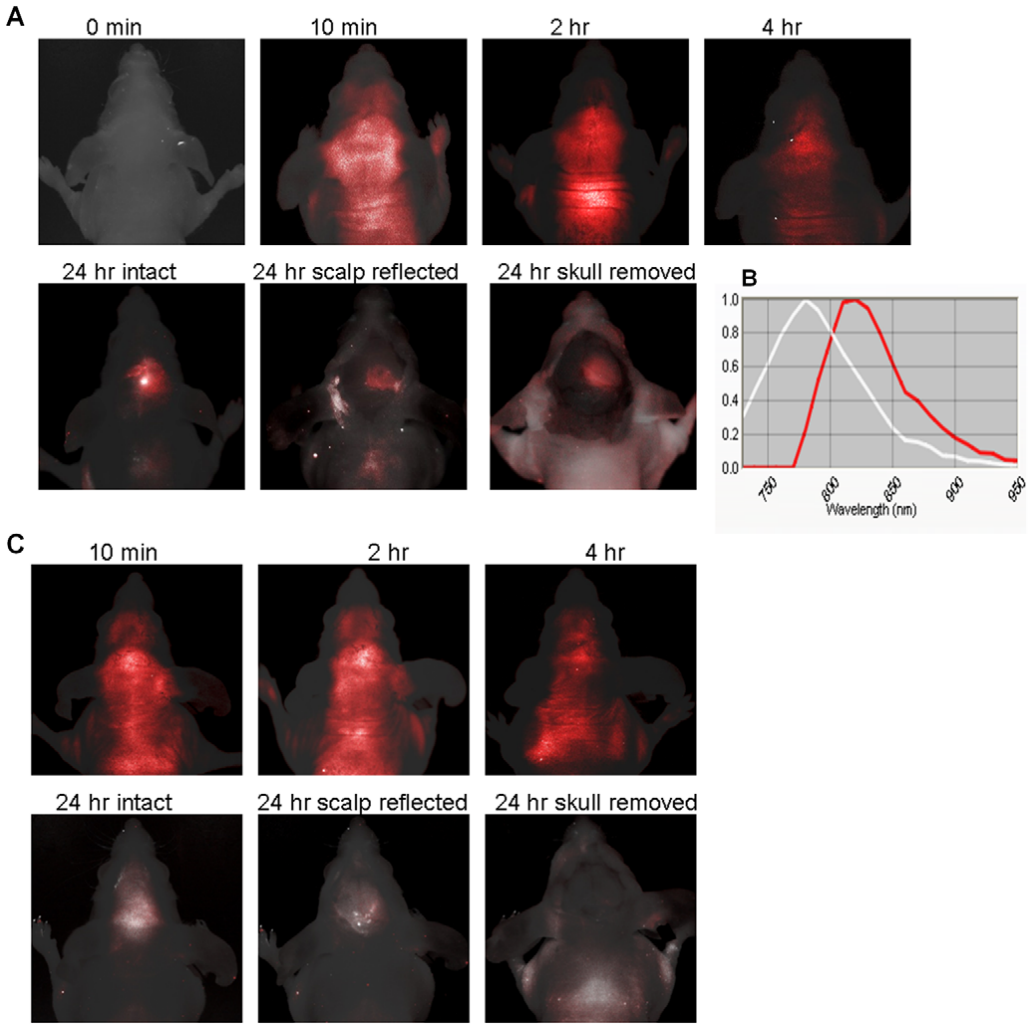
Dynamic *in vivo* fluorescence imaging of the 2-DG dye uptake by intracranial glioma. **A.** After i.v. injection of IRDye800CW 2-DG repeated *in vivo* fluorescence imaging was performed. During the first four hours, unmixed images showing stronger signal of the dye in the brain area, but there was no obvious contrast between the tumor side of the brain and the normal brain. However, 24 h later, the light signal remained only in the tumor side of the intact brain. Even better contrast was seen after reflection of the scalp or further removal of the skull. **B.** Normalized emission spectra showing the near infrared 2-DG dye with a peak emission wavelength at 810 nm (red), while the background signal (white) was at ∼770 nm. **C.** As a control of the 2-DG dye, IRDye800CW carboxyl was injected into a mouse bearing orthotopic glioma. No significant difference in light intensity between the two sides of the brain was observed at any time points.

**Figure 4:**
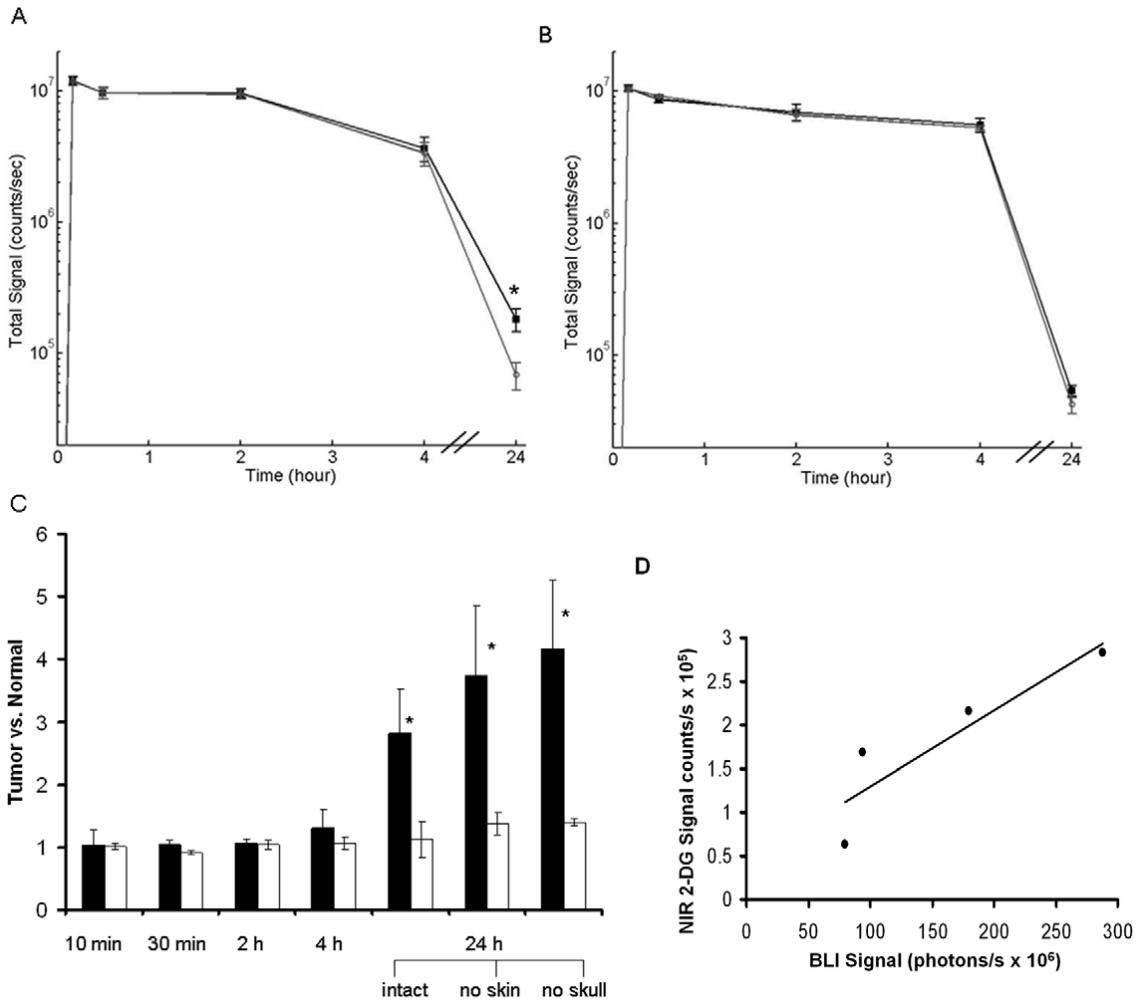
Time course of light intensity of *in vivo* fluorescence imaging of the 2-DG dye. Mean light intensity curves shown for the tumor side (solid square) and the contralateral normal brain (open circle) of intact mice. **A.** For the 2-DG dye (n = 4), the curves were essentially identical for the first 4 h post injection, but after 24 h, there was a significant difference (1.8×10^5^±0.2×10^5^ versus 0.7×10^5^±0.4×10^5^; p<0.05). **B.** In contrast, the control carboxylated dye showed no difference during the whole course (n = 3). **C.** Time course of tumor to normal tissue ratio (TNR) showing a significantly higher TNR in the 2-DG dye group (mean  = 2.8±0.7; solid bar) versus the control group of the control dye (n = 3; 1.1±0.3; open bar) 24 h after injection (p<0.05). An even bigger TNR was obtained in the 2-DG group by removing the scalp (3.7±1.1) or skull (4.1±1.1). **D.** A strong linear correlation was observed between light intensity of *in vivo* BLI and 2-DG NIR imaging of intracranial tumors (r = 0.9).

**Figure 5:**
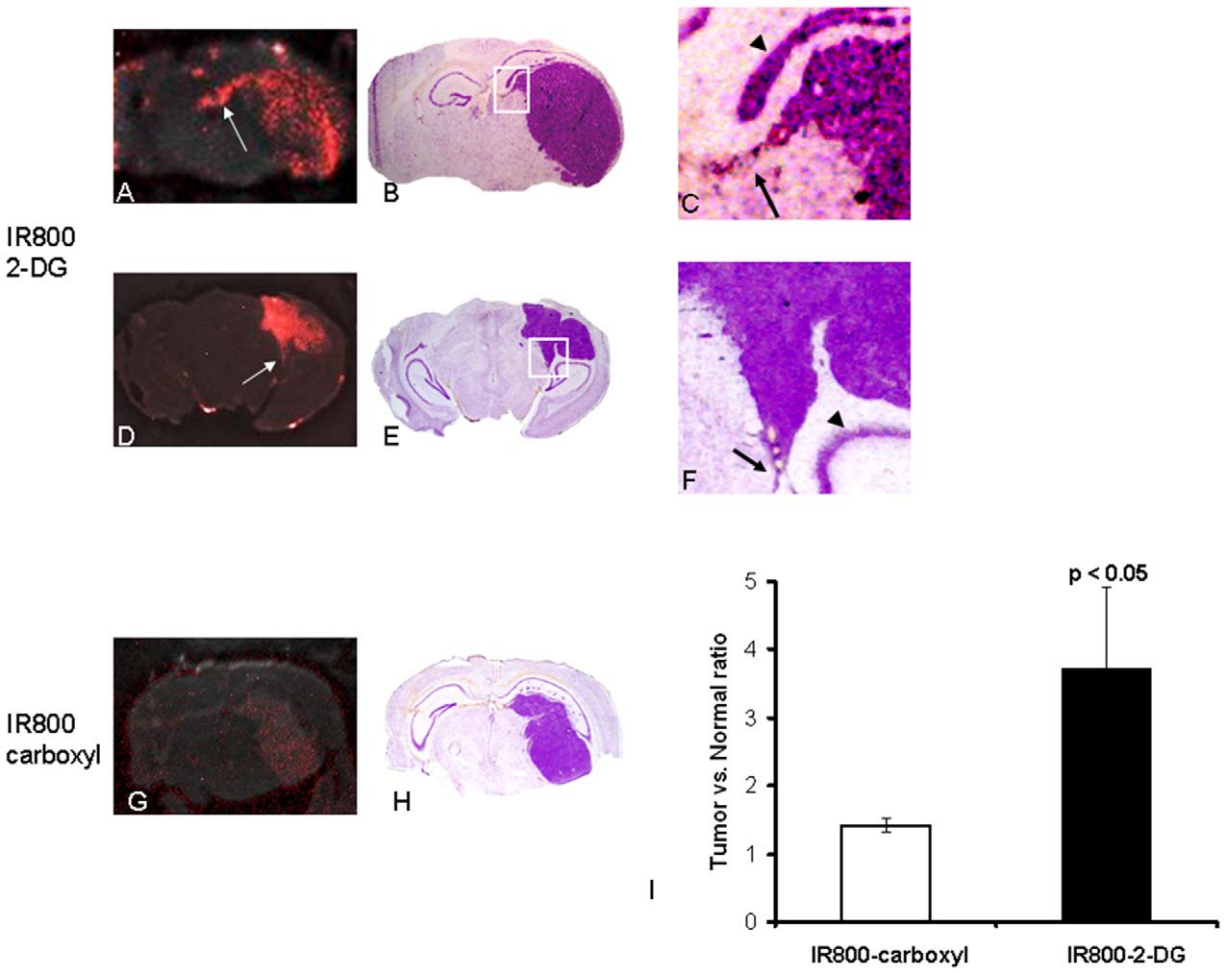
*Ex vivo* near infrared fluorescence imaging of ultrathin cryosections of tumor bearing brain tissues. Unstained coronal brain sections (20 µm) containing tumor tissues were obtained immediately after the 24 h *in vivo* image for *ex vivo* imaging. For the tumors with 2-DG dye, representative images for a larger (A) and a small (D) intracranial tumor showed predominant accumulation of the dye within the tumor mass. Tumor mass was clearly delineated from the surrounding normal tissues. Even a track of migrated tumor cells was depicted in each case (arrows in A and D), which correlated well with cresyl violet staining of the corresponding regions enlarged from B and E (arrows in C and F). Arrow heads refer to the dentate gyrus. The control dye showed no significant difference between the tumor regions and the normal brain (G and H). Tumor/normal ratio (TNR) of the 2-DG dye was significantly higher than that of the control dye (3.7±1.2 vs. 1.4±0.1; p<0.05; I).

**Figure 6:**
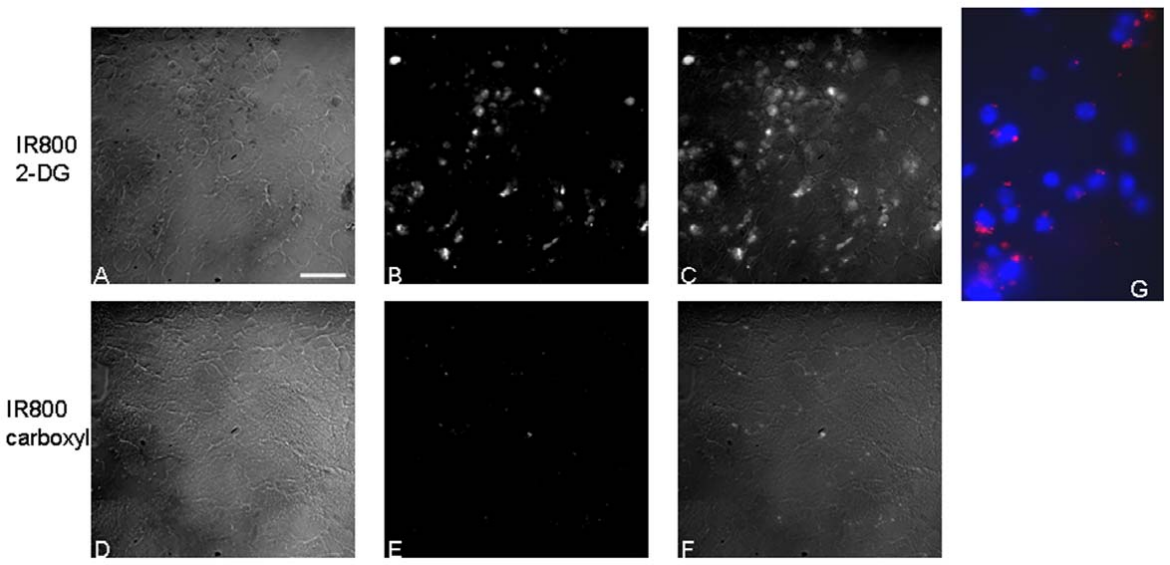
Microscopic fluorescence imaging determined localization of the 2-DG dye. The unstained frozen sections of tumor bearing brain tissues, also used in Fig. 5, were scanned under dark field (A and D). Near infrared signals (B and E) of the same fields detected with an infrared filter were overlaid on the dark field images (C and F). Significantly higher signal was seen in tumor tissues of the mouse injected with the 2-DG dye, compared to the one with control dye. Co-staining of nuclei of tumor cells with DAPI indicated cytoplasmic localization of the 2-DG dye (red; G).

The real-time whole body NIR optical imaging, acquired immediately after i.v. injection of the 2-DG dye, IRDye 800CW 2-DG (15 nmol/mouse) or the control dye, IRDye 800CW carboxylate (15 nmol/mouse), visualized the first pass of the dye sequentially through various organs and tissues (Fig. 2). By narrowing the field of view (FOV), brain-focused *in vivo* imaging showed dynamic changes in fluorescence signal intensity on the tumor side of the brain (right) in comparison with the contralateral normal brain (left). Relatively higher signal intensity was observed for the 2-DG dye in both sides of the brain than the rest of the mouse body (Fig. 3). Time course curves showed that the signal intensity in both sides of the brain had a maximum value of 1.2×10^7^±0.2×10^7^ counts/s 10 min after injection, and gradually washed out over 4 h. There was no significant difference in signal intensity between the two sides of the brain during the first 4 h after injection (Figs. 3A and 4; p>0.3). Twenty four hours later, a dramatic drop in signal intensity was observed. However, significantly higher light signal was clearly seen in the tumor side of the brain than the contralateral normal side (1.8×10^5^±0.2×10^5^ versus 0.7×10^5^±0.4×10^5^; p<0.05; Fig. 4A). A mean tumor/normal ratio (TNR) for the group of 4 tumors was 2.8±0.7 (Fig. 4B). In contrast, the control dye showed little difference (0.54×10^5^±0.1×10^4^ versus 0.43×10^5^±0.3×10^4^; TNR  = 1.1±0.3; Fig. 4B and C). To confirm that the signal originated from the brain tumor, we performed surgical procedures to reflect scalp and further remove skull to expose the brain. A brighter and more focused light signal emitted from the region of the tumor implantation was seen (Fig. 3A). Stronger contrast was achieved by reflecting the scalp (TNR  = 3.7±1.1) and further removal of the skull of the mouse revealed a highest TNR of 4.2±1.1 (Fig. 4C). For the control dye, similar to the observation in the intact animals, no significant difference was found (TNR  = 1.4±0.2 and 1.4±0.1, respectively; Fig. 4C). Comparison of *in vivo* BLI and 2-DG fluorescence imaging of intracranial tumors also showed a strong linear correlation albeit in a small number of animals (n = 4; r = 0.9; Fig. 4D). After sacrificing the mice, the whole brain was dissected and ultrathin sections (20 µm) of tumor bearing brain were imaged using the same fluorescence imaging system as used for *in vivo*. Most intriguingly, distinct tumor margins, even the tracks of migration of small number of tumor cells, were identified based on the fluorescence images, which correlated well with histological staining of the tumor (Fig. 5). In good agreement with *in vivo* imaging data, uptake of the 2-DG dye was found to be significantly higher in tumor than the contralateral normal brain (TNR  = 3.7±1.1; p<0.01; Fig. 5I). Finally, fluorescence microscopy showed more uptake of the 2-DG dye in tumor regions, compared to the control dye (Fig. 6). Co-registration of the NIR signal with the DAPI stained nuclei showed a cytoplasmic localization of the 2-DG dye (Fig. 6G).

## Discussion

We have demonstrated the feasibility of using the NIR dye labeled 2-deoxyglucose, IRDye800CW 2-DG, for *in vivo* fluorescence imaging of orthotopic glioma in a mouse model (Fig. 3). *Ex vivo* fluorescence imaging of tumor sections and microscopic imaging confirmed significantly higher accumulation of the 2-DG dye in intracranial tumors than in normal brain. Good correlations were found between each imaging modality in terms of *in vivo* evaluation of intracranial tumor burden (Figs. 1 and 4D).

Small animal imaging has been increasingly adapted for preclinical cancer research. *In vivo* imaging promises greater efficiency since each animal serves as its own control and multiple time points can be examined sequentially. In particular, multimodal imaging approaches provide comprehensive information about both tumor anatomy and pathophysiology and even molecular mechanisms [4], [20]–[23]. In this study, we combined optical imaging (bioluminescence and fluorescence imaging) with MRI to study longitudinal development of intracranial tumors and their uptake of a glucose analogue, 2-DG. There was good agreement between increased BLI signal intensity over time and enlarged tumor volume measured by MRI, as also reported by others previously for intracranial tumors in rodent models [4], [24]. Thus, the cheap, fast and high-throughput BLI seems to be just as effective in monitoring the deep-seated orthotopic tumor models as the expensive and time consuming MRI. However, BLI does not provide anatomic details due to the poorer spatial resolution and limited depth of light penetration. Recent progress in BLI tomography should facilitate three-dimensional analysis. In addition to providing detailed anatomic structure, MRI is also useful to obtain pathophysiological information, *i.e.*, tumor vascular perfusion and permeability, oxygenation, and apoptosis or necrosis, though not reported here.

Fluorescence imaging can be used to evaluate small reporter molecule pharmacokinetics, avoiding the need for genetic modification of cells. In particular, near infrared fluorescence (NIR) imaging has found a greater potential for clinical application because of its long wavelength (650–900 nm), where light absorbance and scattering are significantly lower, and autofluorescence of normal tissues is also greatly reduced [25]. In view of this approach, various target-specific NIR conjugates have been reported for targeting tumor imaging, *e.g.*, tumor integrin, α_v_β_3_ [4], tumor growth factors or their receptors [18], [26]–[30], glycoprotein [31], or tumor specific protease [21]. Successful applications in various preclinical tumor models have been reported, though most of these studies were performed on surface tumors. *In vivo* NIR imaging of deep-seated orthotopic tumor models, in particular, intracranial tumors, remains challenging. By targeting the overexpressed α_v_β_3_ in tumor, Hsu, *et al.* recently reported visualization of orthotopic glioma of mouse *in vivo* by NIR imaging via Cy5.5-RGD. Peak of tumor uptake was found 2 h post injection and TNR  = 2.6 was achieved with a craniotomy [4]. More recent work with an orthotopic mouse brain tumor McCann, *et al.* successfully applied fluorescent molecular tomographic imaging to monitor protease activity in tumor by using protease activatable fluorescence, ProSense680 (peak light emission at 680 nm). By co-registering fluorescence images with MRI, localization of the active protease in tumor was determined [5].

Here, we applied the commercially available NIR labeled 2-deoxyglucose to imaging intracranial tumors based on the simple mechanism of differential levels of glucose metabolism between tumors and normal tissues. Tumor cells both require more energy for their higher proliferation rate and utilize the inefficient glycolytic pathway to produce energy. Thus, tumor cells need more glucose compared to normal cells. On this basis, ^18^FDG has been used as the most common PET radiotracer to visualize clinical tumors and their metastases. However, ^18^FDG PET imaging of brain tumor is often compromised by strong background signals of normal brain tissues. Due to the short half life of fluorine-18 (<2 h), PET imaging is normally performed within 1 h after injection of ^18^FDG. The lower contrast between tumor and normal brain may be partly attributed to this timing, at which a maximum ratio of uptake is not reached. Indeed, we found no significant difference in light signal in the tumor side of the brain versus the normal side of the brain during the first 4 hours post injection. The peak tumor to normal ratio was actually observed at 24 h, which is consistent with a previous study of subcutaneous U87 tumors using cy5.5 labeled D-glucosamine. However, a control dye, cy5.5-NHS used in that study also produced a high TNR [16]. Our study is also in a good agreement with another study that detected essentially no retention of IRDye800CW 2-DG in normal brain after 24 h assessed by *ex vivo* imaging [19]. In addition to tumor diagnostic imaging, pyropheophorbide labeled 2-deoxyglucosamine has shown a potential for photodynamic therapy on tumors [32], [33].

Our results of *ex vivo* fluorescence imaging showed that the 2-DG dye distributed well into the whole tumor despite some heterogeneity (Fig. 5). This observation may suggest delivery and distribution of the 2-DG dye to the tumor does not need the BBB disruption, which is the prerequisite condition for the dyes currently used for neurosurgery. Indeed, uncoupling of tumor vascular perfusion and permeability and uptake of FDG has been reported previously [35], [36]. However, it is still possible that the dye first leaks out through the disrupted BBB, from which it diffuses into the whole tumor. Further studies will be necessary using earlier stage of tumors, when the Gd contrast leakage is not obviously seen by T_1_-weighted contrast enhanced MRI, to prove this hypothesis. Furthermore, the usefulness of the 2-DG dye to stain the infiltrative tumor cells is limited by the tumor model used in this study. The U87 tumor has relatively sharp tumor-brain boundaries. GBM models showing more aggressiveness will be needed to test the ability to stain the finger-shaped infiltrative tumors. Instead of the established GBM cell lines, using surgically resected tumor tissues directly from GBM patients and passing them in animals will generate stable orthotopic GBM xenografts, which show oncogene patterns very similar to primary tumors of patients.

In summary, fluorescence imaging of deoxyglucose uptake in more clinically relevant orthotopic glioma models has not yet been reported. Our results may suggest an optimal time for imaging brain tumors based on the glucose analogues. The near-infrared dye labeled 2-DG may serve as a novel imaging probe to noninvasively monitor intracranial tumor burden in preclinical animal models. From a clinical perspective, development of NIR labeled tumor-specific molecules may have the potential to identify the infiltrating glioblastoma intra-operatively, and to improve the extent of resection of selected tumors.

## Materials and Methods

### Orthotopic Glioma Xenografts

All animal procedures were approved by the Institutional Animal Care and Use Committee of University of Texas Southwestern Medical Center. Human glioma U87 MG cells (ATCC, Manassas, VA, USA) were infected with a lentivirus containing a firefly luciferase reporter, and highly expressing stable clones were isolated. A ∼1 cm long incision of skin was made along the midline of the brain of an anesthetized nude mouse (BALB/c nu/nu; Harlan, Indianapolis, IN). Using a high-speed drill, a 1 mm burr hole of the skull was made in the right hemisphere, anterior to the coronal fissure. About 5×10^4^ U87-luc cells in 3 µl mixture of PBS and Matrigel (25%, BD Biosciences, San Jose, CA) were injected directly into right caudal diencephalon, 1.5 mm beneath the dura mater using a 32G Hamilton syringe. Usage of a 32G fine needle minimizes tissue damage. The burr hole was filled with bone wax and the scalp was closed with sutures.

### *In Vivo* BLI

BLI was initiated for monitoring intracranial tumor growth about 10 days after tumor implantation and repeated once a week using the IVIS Spectrum system (Caliper, Xenogen, Alameda, CA). The tumor bearing mice (n = 7) were anesthetized (isoflurane/O_2_ in an induction chamber; isoflurane from Baxter International Inc., Deerfield, IL) and a solution of *D*-luciferin (120 mg/kg in PBS in a total volume of 80 µl; Biosynthesis, Naperville, IL) was administered s.c. in the neck region. Anesthesia was maintained with isoflurane (2%) in oxygen (1 dm^3^/min). Five minutes after luciferin injection, an array of various exposure time (1, 5, 30, 60 s) was applied for image acquisition. Data were quantified with the Living Imaging software by using absolute photon counts (photons/s) in an ROI, manually drawn to outline the BLI signal of the brain.

### *In Vivo* MRI

Once a BLI signal was observed, MRI was performed to assess tumor volume and growth by using a 4.7 T horizontal bore magnet with a Varian INOVA Unity system (Palo Alto, CA). Each mouse was maintained under general anesthesia (air and 2% isoflurane). A 27 G butterfly (Abbott Laboratories, Abbott Park, IL) was placed in a tail vein for contrast agent administration. Pertinent image slice positions were based on fast sagittal scout images. T_1_-weighted (TR  = 250 ms; TE  = 20 ms; slice thickness  = 1.5 mm; FOV  = 25×25; in plane resolution 195 µm) and corresponding T_2_-weighted (TR  = 1900 ms; TE  = 80 ms) spin-echo multislice coronal images were acquired. T_1_-weighted contrast enhanced images were acquired after i.v. bolus injection of the contrast agent Gd-DTPA-BMA (0.1 mmol/kg body weight; Omniscan^TM^, Amersham Health Inc., Princeton, NJ) through the tail vein catheter. We determined tumor volume on T_2_-weighted images by manually outlining the enhancing portion of the mass, excluding congested CSF signal in ventricles, on each image by using standard “browser” software provided with the Varian Inova imaging system. The area measurements were automatically calculated and multiplied by the MRI section thickness to calculate a per-section tumor volume. The total tumor volume was obtained by summing the volume calculations for all sections.

### Near Infrared Fluorescence Imaging

#### *In vivo* real-time imaging of first pass perfusion

When the intracranial tumors grew to larger than 4 mm in diameter based on T_2_-weighted MRI, *in vivo* fluorescence imaging was performed using a Maestro imaging system (CRI Inc. Woburn, MA). Each mouse was anesthetized by i.p. injection of a ketamine/xylazine cocktail (40 µl). A 27 G butterfly (Abbott Laboratories) was placed in a tail vein for administration of the 2-DG dye, IRDye800CW 2-DG (15 nmol/mouse in 150 µl saline, Li-Cor Biosciences, Lincoln, NB) or a control dye, IRDye800CW carboxylate (15 nmol/mouse in 150 µl saline, Li-Cor Biosciences). Based on the methodology developed by Hillman and Moore [37], an excitation filter (671–705 nm) was applied. A series of whole body images was acquired before and after a bolus injection of the 2-DG dye or the control dye. Typically, 5 frames/s for the first 20 s, 2 frames/s for the next 30 s and 1 frame/s for the rest of ∼2 min were acquired. Image processing and data analysis were performed using the DyCE software provided with the Maestro software 2.8.

#### *In vivo* dynamic imaging of 2-DG uptake by intracranial tumors

The brain-focused fluorescence images were acquired before and 10 min, 30 min, 2 h, 4 h, and 24 h after injection of the 2-DG or the control dye. A set of filters specifically for NIR imaging (excitation, 671–705 nm; emission, 730–950 nm) was applied. At each time point, the mouse was anesthetized by i.p. injection of the ketamine/xylazine cocktail (40 µl). Immediately after the last image at 24 h, the scalp of the mouse was surgically reflected to expose the skull and a fluorescence image was acquired. Before sacrificing the mouse, the skull was removed to expose both sides of the brain tissues and a last *in vivo* image was obtained. The whole surgical procedure and imaging was completed within 15 min on the anesthetized animals and no obvious bleeding was observed.

#### *Ex vivo* fluorescence imaging on cryosections of tumor bearing brain

Immediately after sacrificing the animals, the whole brains were dissected and embedded in O.C.T. and then transferred to −80°C freezer. On the second day, a series of coronal sections (20 µm) of the brain was cut. The frozen sections containing tumor tissues were identified by cresyl violet staining. The adjacent tissue sections were used for *ex vivo* fluorescence imaging and fluorescence microscopic study, as described in detail below. The *ex vivo* images were taken by using the same Maestro system as used for *in vivo* studies. Typically, an exposure time of 2.5 s was applied for image acquisition.

### Analysis of Fluorescence Imaging

Fluorescence images were obtained using the Maestro system and processed with the Maestro software 2.8. The spectrum of background signal (peak emission ∼770 nm) was first obtained from a mouse before the IRDye 800cw dye injection, while the spectrum of IRDye 800cw dye (peak emission ∼810 nm) was detected from a solution of the dye in PBS (0.1 nmol/µl). The spectra were then imported and used to unmix the NIR dye signal from the background signal for both *in vivo* and *ex vivo* studies. The whole set of *in vivo* images of an individual mouse, obtained before and at various times after injection of the dye, was examined for quantification. A common ROI was based on the most obvious signal of the tumor, which was observed on the image of the skull-deprived mouse 24 h after injection in each of the 2-DG animals, was applied on both the tumor side and the contralateral normal brain of each image. A total of photon counts (counts/s) in the identical ROI was used for comparison of dynamic change in signal intensity.

### Histology and Fluorescence Microscope

Cresyl violet staining was performed on the cryosections (20 µm). Tumor margins were determined under microscope and correlated with the NIR light signal acquired by *ex vivo* fluorescence imaging. The cryosections, unstained or counterstained by 4′,6-diamidino-2- phenylindole (DAPI), were used for fluorescence microscopy study. The NIR fluorescence signal was detected using a Zeiss AxioObserver (Carl Zeiss MicroImaging, Inc., Thornwood, NY) equipped with NIR filters. The NIR signals were recorded and overlaid on either a dark image or an image counterstained by DAPI of the same field. For luciferase staining, monoclonal mouse antiluciferase mAb (1∶150; AbD Serotec, Raleigh, NC) and cy3-conjugated goat anti-mouse secondary antibody (Jackson Immunoresearch Laboratories, West Grove, PA) were used.

### Statistical Analysis

Statistical significance was assessed using an ANOVA on the basis of Fisher's protected least significant difference (PLSD; Statview; SAS Institute Inc., Cary, NC) or Student's t tests.
